# Remarks on the Diet of Children, &c.

**Published:** 1847-07-01

**Authors:** 


Remarks on the Diet of Children; and on the Distinctions between the Digestive Powers of the Infant and the Adult. By *George T. Gream,* one of the principal Medical Officers of the Queen Charlotte's Lying-in Hospital, &c. &c. Small 8vo, pp. 201. London: Longman and Co., 1847.

We should have been quite at a loss to know why Mr. Gream should have published this book, if he had not been so obliging as, in the concluding paragraph of it, to assign his reasons for doing so.

"In bringing to a conclusion," he says, "these practical remarks, which have been almost extorted from me by the sufferings to which numbers of children are exposed, as I daily witness, through the ignorance or thoughtlessness of their attendants, I venture to express a hope that they may not only prove useful, in the domestic circle, among parents, and others who have the charge of infancy and youth, but that they may, as tending in however humble a degree, to advance and enforce their own views and principles, obtain a favourable reception from those of my own profession, to whom we are indebted for the establishment, on scientific grounds, of those practical and judicious regulations for the management of children, which are now more or less adopted by the most eminent and successful practitioners." P. 201.

We fear, from the loose, superficial, and common-place character of Mr. Gream's observations, that his most alluring motive was the not very disinterested one of being useful in the *domestic circle, among parents, and others who have the charge of youth.* We think it just possible that, in this respect, the book may answer the purpose; because the persons to whom it is addressed are not the most discriminating critics of a medical author's merits, and Mr. Gream has several very comforting precedents of similar popular books having puffed their authors into practice. After these remarks we need scarcely say that, to well-educated medical men, or even to pupils, Mr. Gream's book is not likely to be of use; and that it is more likely to be a vade-mecum for Mr. Gream himself, than to have any more extensive application.

